# Contemporary Circulating Enterovirus D68 Strains Have Acquired the Capacity for Viral Entry and Replication in Human Neuronal Cells

**DOI:** 10.1128/mBio.01954-18

**Published:** 2018-10-16

**Authors:** David M. Brown, Alison M. Hixon, Lauren M. Oldfield, Yun Zhang, Mark Novotny, Wei Wang, Suman R. Das, Reed S. Shabman, Kenneth L. Tyler, Richard H. Scheuermann

**Affiliations:** a J. Craig Venter Institute, Rockville, Maryland, USA; b Neuroscience Program and Medical Scientist Training Program, University of Colorado School of Medicine, Aurora, Colorado, USA; c J. Craig Venter Institute, La Jolla, California, USA; d Department of Neurology, University of Colorado School of Medicine, Aurora, Colorado, USA; e Denver VA Medical Center, Denver, Colorado, USA; f Department of Immunology and Microbiology, University of Colorado School of Medicine, Aurora, Colorado, USA; g Department of Medicine, University of Colorado School of Medicine, Aurora, Colorado, USA; h Department of Pathology, University of California, La Jolla, California, USA; Johns Hopkins Bloomberg School of Public Health; National Institutes of Health; Utah State University

**Keywords:** EV-D68, SH-SY5Y, enterovirus, myelitis, neurotropism, neurovirology

## Abstract

Enterovirus D68 (EV-D68) has historically been associated with respiratory illnesses. However, in the summers of 2014 and 2016, EV-D68 outbreaks coincided with a spike in polio-like acute flaccid myelitis/paralysis (AFM/AFP) cases. This raised concerns that EV-D68 could be the causative agent of AFM during these recent outbreaks. To assess the potential neurotropism of EV-D68, we utilized the neuroblastoma-derived neuronal cell line SH-SY5Y as a cell culture model to determine if differential infection is observed for different EV-D68 strains. In contrast to HeLa and A549 cells, which support viral infection of all EV-D68 strains tested, SH-SY5Y cells only supported infection by a subset of contemporary EV-D68 strains, including isolates from the 2014 outbreak. Viral replication and infectivity in SH-SY5Y were assessed using multiple assays: virus production, cytopathic effects, cellular ATP release, and VP1 capsid protein production. Similar differential neurotropism was also observed in differentiated SH-SY5Y cells, primary human neuron cultures, and a mouse paralysis model. Using the SH-SY5Y cell culture model, we determined that barriers to viral binding and entry were at least partly responsible for the differential infectivity phenotype. Transfection of genomic RNA into SH-SY5Y generated virions for all EV-D68 isolates, but only a single round of replication was observed from strains that could not directly infect SH-SY5Y. In addition to supporting virus replication and other functional studies, this cell culture model may help identify the signatures of virulence to confirm epidemiological associations between EV-D68 strains and AFM and allow for the rapid identification and characterization of emerging neurotropic strains.

## Abstract (precis)

Since the EV-D68 outbreak during the summer of 2014, evidence of a causal link to a type of limb paralysis (AFM) has been mounting. In this article, we describe a neuronal cell culture model (SH-SY5Y cells) in which a subset of contemporary 2014 outbreak strains of EV-D68 show infectivity in neuronal cells, or neurotropism. We confirmed the difference in neurotropism *in vitro* using primary human neuron cell cultures and *in vivo* with a mouse paralysis model. Using the SH-SY5Y cell model, we determined that a barrier to viral entry is at least partly responsible for neurotropism. SH-SY5Y cells may be useful in determining if specific EV-D68 genetic determinants are associated with neuropathogenesis, and replication in this cell line could be used as rapid screening tool for identification of neurotropic EV-D68 strains. This may assist with better understanding of pathogenesis and epidemiology and with the development of potential therapies.

## INTRODUCTION

The Enterovirus genus in the Picornaviridae family comprises many important human pathogens, including the following: human rhinoviruses (HRVs), the most common viral agents of the common cold; polioviruses, the causative agent of poliomyelitis; enterovirus A71 (EV-A71), associated with a variety of neurological diseases; and enterovirus D68 (EV-D68). Enteroviruses appear to continually circulate in human populations, with most infections being asymptomatic. For example, up to 72% of poliovirus infections are asymptomatic (1). When poliovirus infections are symptomatic, they can cause a wide spectrum of clinically distinct syndromes, ranging from minor, nonspecific illness, to nonparalytic aseptic meningitis and flaccid paralysis (2). Before the widespread use of effective vaccines, poliovirus-induced paralysis reached a peak of 21,000 cases in the United States in 1952 (3).

EV-D68 was first detected in children with pneumonia and bronchiolitis in 1962 (4). Until recently, EV-D68 was one of the most rarely reported enteroviruses, with only 26 cases documented by the National Enterovirus Surveillance System in the United States from 1970 to 2005 (5). Beginning in 2009, multiple contemporary clades began emerging worldwide (6). In the summer and fall of 2014, 49 U.S. states experienced a nationwide outbreak of severe respiratory illness associated with EV-D68, with 1,153 confirmed cases, including 14 deaths (7). Shortly after the U.S. outbreak, EV-D68 infections were also reported in Canada, Europe, and Asia. The total number of reported EV-D68 cases in 2014 exceeded 2,000 from 20 countries, resulting in the public health community classifying EV-D68 as a reemerging pathogen of public health concern (8).

Reports of acute flaccid myelitis (AFM) occurring coincident to the outbreak of EV-D68 respiratory disease raised the possibility that EV-D68 might be a causative agent of AFM (7). EV-D68 infection within a subset of these AFM cases was confirmed in several independent epidemiological clusters in the United States (9–14), France (15), Norway (16), Canada (17), and Australia (18). Statistical analyses of the AFM cases in Colorado (12) and California (19) have supported the association between EV-D68 and AFM, and viral nucleic acid detection studies of patient samples have failed to reveal an alternative etiology (7, 10). During the 2014 EV-D68 outbreak, patients presenting with AFM showed distinctive magnetic resonance imaging (MRI) findings characterized by brain stem and gray matter longitudinally extensive spinal cord lesions. This matches the findings described in previous outbreaks of EV-A71-associated AFM (9, 19, 20), suggesting that an enterovirus may be responsible. In support of this hypothesis, Hixon et al. (21) established that several contemporary EV-D68 strains, but not the historically archetypal Fermon and Rhyne EV-D68 strains, can cause a paralytic disease in neonatal mice due to viral infection and killing of spinal cord motor neurons.

Phylogenetic analysis reported that many of the 2014 EV-D68 outbreak isolates associated with AFM appeared to belong to the phylogenetic subclade B1 (10, 22). Interestingly, 12 substitutions identified in B1 2014 isolates carry the same amino acid or nucleotide residues observed at equivalent positions in other paralysis-causing enteroviruses, including poliovirus and EV-A71 (22). This suggests that one or more of the nucleotide substitutions present in contemporary EV-D68 strains and lineages and not found in historical archetypal strains may be responsible for the apparent increased incidence of neuropathology associated with the 2014 outbreak. EV-D68 has continued to evolve since the 2014 outbreak, which is unsurprising as mutation and recombination are known to occur in enteroviruses (23–25). Sequence analysis has led to the classification of a new clade, D (a subclade of A) (26, 27), and a new subclade, B3, has emerged and quickly expanded (26, 28, 29). Neurological symptoms have been associated with the novel B3 clade in Sweden (30), The Netherlands (31), Taiwan (32), Italy (33), and the United States (34), which experienced another AFM outbreak during the 2016 enterovirus season (summer and fall), with a total of 149 confirmed cases (2). The seasonality and magnitude of this AFM outbreak match the AFM surge observed in 2014. Additional surveillance of potentially emerging neurotropic or neuropathogenic strains is warranted.

To test if a specific genotype is associated with neurological symptoms, we report the development of a cell culture infection model based on the neuroblastoma cell line SH-SY5Y, which shows differential infectivity by different EV-D68 isolates. We observe a correlation between infection and replication in SH-SY5Y cells and neuropathogenesis in mice. This neuronal SH-SY5Y model may be useful for analysis of virus-host interactions *in vitro* and provides a facile assay to quantify which EV-D68 strains are neurotropic and neuropathogenic, potentially leading to better surveillance of virulent EV-D68 strains.

## RESULTS

### SH-SY5Y cells express higher levels of neuron-specific genes than other candidate cell lines.

Seeking a human cell culture to model neuron-specific infectivity, we performed transcriptome profiling of two commonly used “neuronal-like” cell lines, SH-SY5Y and HTB10. These cell lines were compared with HeLa cells as a nonneuronal permissive cell culture model. Both of these neuronal cell lines were first cultured by Biedler et al. in the early 1970s. SH-SY5Y is a subclone of the HTB11 (SK-N-SH) neuroblastoma cell line that was selected as an apparently homogenous population of cells with neuronal cell morphology. HTB10 (also known as SK-N-MC), reported as a neuroepithelioma cell line, has been used as a model for different neurotrophic viruses, such as hepatitis C poliovirus (35, 36) and enterovirus A71 (37). We used RNA sequencing (RNA-seq) to determine the genes expressed in SH-SY5Y, HeLa, and HTB10 cells and specifically assessed the expression of 30 neuronal cell marker genes that were selected as being highly neuron specific (Table 1). Of the 30 selected genes, 24 showed measurable expression in SH-SY5Y cells, 23 of which showed higher expression levels in SH-SY5Y compared to HTB10 cells, and little if any expression in HeLa cells. These findings support the use of SH-SY5Y as a model neuronal cell line, while raising questions about the suitability of HTB10 as a “neuronal-like” cell line.

**TABLE 1:** Expression of neuron-specific genes in HeLa, HTB10, and SH-SY5Y cell lines

Gene	Gene product name	Expression (TPM) in line^*a*^:		
		HeLa	HTB10	SH-SY5Y
*STMN2*	Stathmin 2	0.0	0.0	1,322.5
*TCEAL7*	Transcription elongation factor A-like 7	0.0	0.0	248.5
*RGS4*	Regulator of G-protein signaling 4	0.0	0.0	163.7
*ISL1^*b*^*	Insulin gene enhancer protein 1	0.0	0.0	55.7
*NNAT*	Neuronatin	0.0	0.0	45.8
*VIP*	Vasoactive intestinal peptide	0.0	0.0	39.3
*TAGLN3*	Transgelin 3	0.0	0.0	39.0
*SNAP25*	Synaptosome-associated protein 25	1.8	0.7	24.6
*LMO1*	LIM domain only 1	0.6	0.0	17.0
*CAMK2N1*	Calmodulin-dependent protein kinase II inhibitor 1	1.2	0.0	11.8
*SCG2*	Secretogranin II	0.0	0.0	10.7
*CDO1*	Cysteine dioxygenase type 1	0.0	0.0	6.9
*SLC10A4*	Solute carrier family 10 member 4	0.0	0.0	3.1
*DLX5*	Distal-less homeobox 5	0.0	0.0	2.1
*DBH*	Dopamine β-hydroxylase	0.0	0.0	1.7
*SYT17*	Synaptotagmin 17	0.1	0.0	1.1
*CUX2*	Cut-like homeobox 2	0.0	0.1	1.1
*CNTN4*	Contactin 4	0.0	0.2	1.0
*DPYSL5*	Dihydropyrimidinase-like 5	0.0	0.2	1.0
*SV2C*	Synaptic vesicle glycoprotein 2C	0.0	0.0	0.7
*ETV1*	ETS variant 1	0.0	0.1	0.7
*DCN*	Decorin	0.0	7.9	0.6
*ISL2^*b*^*	Insulin gene enhancer protein 2	0.0	0.0	0.3
*NXPH1*	Neurexophilin 1	0.0	0.0	0.1
*FOXP2*	Forkhead box P2	0.2	2.2	0.0
*GAD1*	Glutamate decarboxylase 1	0.0	0.2	0.0
*NELL1*	Neural EGFL-like 1	0.0	0.3	0.0
*SLC17A7*	Solute carrier family 17 member 7	0.0	0.6	0.0
*CHAT^*b*^*	Choline acetyltransferase	0.0	0.0	0.0
*OLIG2^*b*^*	Oligodendrocyte transcription factor	0.0	0.0	0.0

a Shown are expression levels (transcripts per million reads [TPM]) of 26 highly neuron-specific marker genes selected from the Allen Brain Atlas (82–84; http://brain-map.org) and BioGPS (85, 86; http://biogps.org) as examined by RNA sequencing.

b Genes selectively expressed in motor neurons were selected from https://en.wikipedia.org/wiki/Neuronal_lineage_marker.

### A representative B1 clade EV-D68 strain, US/MO/47, replicates in SH-SY5Y cells.

Given the epidemiological association of recent EV-D68 infections with AFM, we sought to determine if there are any differential growth phenotypes between contemporary and historical EV-D68 strains in neuronal versus nonneuronal cell lines. To measure viral replication kinetics, each cell line was infected at a multiplicity of infection (MOI) of 0.1, and the virus growth was measured by determining virus titers (50% tissue culture infective dose [TCID_50_]) in culture supernatant at five time points postinfection. To examine whether viruses from the B1 clade showed any phenotypic differences in their ability to infect human neuronal cells, we first selected three different viruses to represent the phylogenetic diversity of EV-D68. US/MO/14-18947 from Missouri (US/MO/47) was selected as a representative of the B1 clade since it carries all 21 substitutions identified in our previous comparative genomics analysis (22). USA/N0051U5/2012 from Tennessee (US/TN) was selected as a representative of clade A since it was isolated in the United States (38) during roughly the same time frame as US/MO/47 and possesses none of the 21 substitutions. VR1197 was selected as an example of an historical isolate similar to the prototypical Fermon strain isolated in 1962.

**FIG 1:**
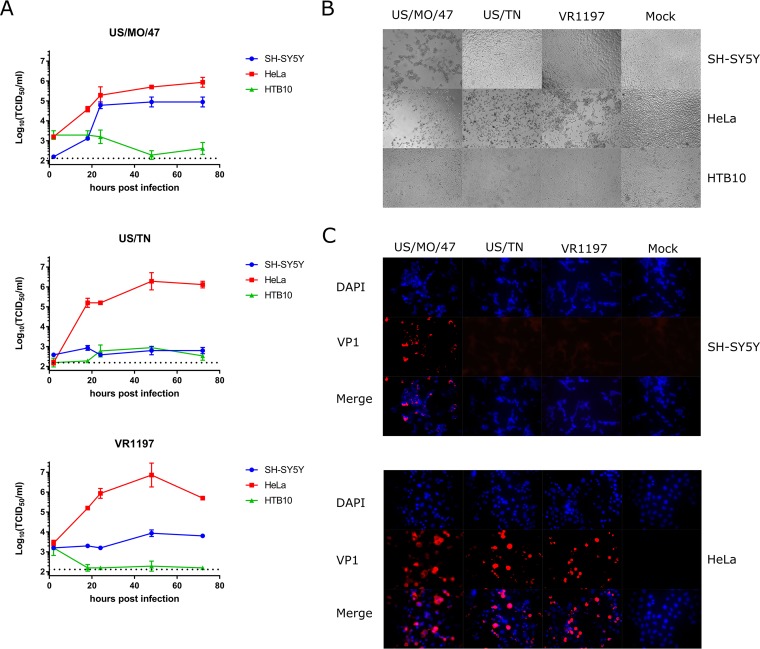
Differential infection and replication of EV-D68 strains in SH-SY5Y. (A) SH-SY5Y, HTB10, and HeLa cells were grown to 90% confluence in 96-well plates before infection with EV-D68 US/MO/47, US/TN, and VR1197 at an MOI of 0.1. Infection medium was removed 2 h postinfection (hpi) to reduce background. Cell culture lysates were collected at various time points after infection, and viral titers were measured using endpoint dilutions for growth in HeLa cells. The dotted black line indicates the limit of detection. Error bars represent standard error of the mean (SEM) from three biological replicates. (B) SH-SY5Y, HTB10, and HeLa cells were infected with EV-D68 US/MO/47, US/TN, and VR1197 at an MOI of 0.1 as described above. Cells were visualized at 72 hpi with bright-field microscopy at 400×. (C) HeLa and SH-SY5Y cells were infected with the indicated EV-D68 strains at an MOI of 1.0. Cells were fixed at 18 hpi and stained with polyclonal antiserum against EV-D68 VP1 (red) and counterstained with DAPI (blue) for detection of nuclei.

All three EV-D68 strains replicate in the nonneuronal cell line HeLa (Fig. 1A) and the alveolar A549 cell line (see Fig. S1 in the supplemental material). These viruses also cause cell death as judged by visual evidence of cytopathic effect (CPE) in infected cell culture (Fig. 1B). In contrast, only US/MO/47 could replicate in the SH-SY5Y neuronal cell line, reaching peak titers of ∼10^5^ TCID_50_/ml by 48 h postinfection (hpi). US/TN and VR1197 did not show any signs of replication, with titers not exceeding background after 96 hpi (Fig. 1A). Similarly, CPE was observed after infection of SH-SY5Y cells with US/MO/47, but not with US/TN or VR1197 (Fig. 1B). Similar results were seen at MOI of 0.01 and 1.0 (see Fig. S2 in the supplemental material). Furthermore, multiple passages of US/TN- or VR1197-infected supernatant onto fresh SH-SY5Y cells (passaging every 4 days for 12 days) failed to produce CPE (data not shown). No increase in viral titers above background levels was detected by any virus following infection of HTB10.

10.1128/mBio.01954-18.1 FIG S1 EV-D68 virus titers in A549 cells. A549 cells were grown to 90% confluence in 96-well plates before infection with EV-D68 US/MO/47, US/TN, and VR1197 EV-D68 at an MOI of 0.1. Infection medium was removed after 2 hpi to reduce background. Cell culture lysates/supernatants were collected at various time points after infection, and viral titers were measured using endpoint dilutions for growth in HeLa cells. The dotted black line indicates the limit of detection. Error bars represent SEM from three biological replicates. Download FIG S1, TIF file, 0.3 MB. Copyright © 2018 Brown et al. 2018 Brown et al. This content is distributed under the terms of the Creative Commons Attribution 4.0 International license.

10.1128/mBio.01954-18.2 FIG S2 EV-D68 virus titers in three different cell cultures with additional MOIs. Cells from three different cell lines—SH-SY5Y, HTB10, and HeLa—were grown to 90% confluence in 96-well plates before infection with EV-D68 US/MO/47, US/TN, and VR1197 EV-D68 at MOI of 1.0 and 0.01. Infection medium was removed after 2 hpi to reduce background from an MOI of 1.0. Cell culture lysates/supernatants were collected at various time points after infection, and viral titers were measured using endpoint dilutions for growth in HeLa cells. The dotted black line indicates the limit of detection. Error bars represent SEM from three biological replicates. Download FIG S2, TIF file, 1 MB. Copyright © 2018 Brown et al. 2018 Brown et al. This content is distributed under the terms of the Creative Commons Attribution 4.0 International license.

### Immunofluorescence confirms US/MO/47 replication in SH-SY5Y cells.

We also examined production of the virus VP1 protein during infection of HeLa and SH-SY5Y cells. VP1 is among the initial proteins expressed following picornavirus infection, preceding capsid assembly (39). We examined the ability of US/MO/47, US/TN, and VR1197 viruses to synthesize the VP1 capsid protein 18 hpi using immunofluorescence. VP1 protein was detected in cells following infection of HeLa cells with all three EV-D68 strains (Fig. 1C). However, only the US/MO/47 isolate produced VP1 following infection of the SH-SY5Y neuronal cell line (Fig. 1C), which is consistent with our data on viral replication and CPE (Fig. 1A and B). Also consistent with the viral replication experiments, no VP1 was produced by any of the three strains when HTB10 cells were infected (data not shown). As typically observed in picornaviruses, VP1 staining was observed in the cytoplasm and not the nucleus of both HeLa and SH-SY5Y cells.

### Intramuscular virus injection of neurotropic EV-D68 causes paralysis in neonatal mice.

**FIG 2:**
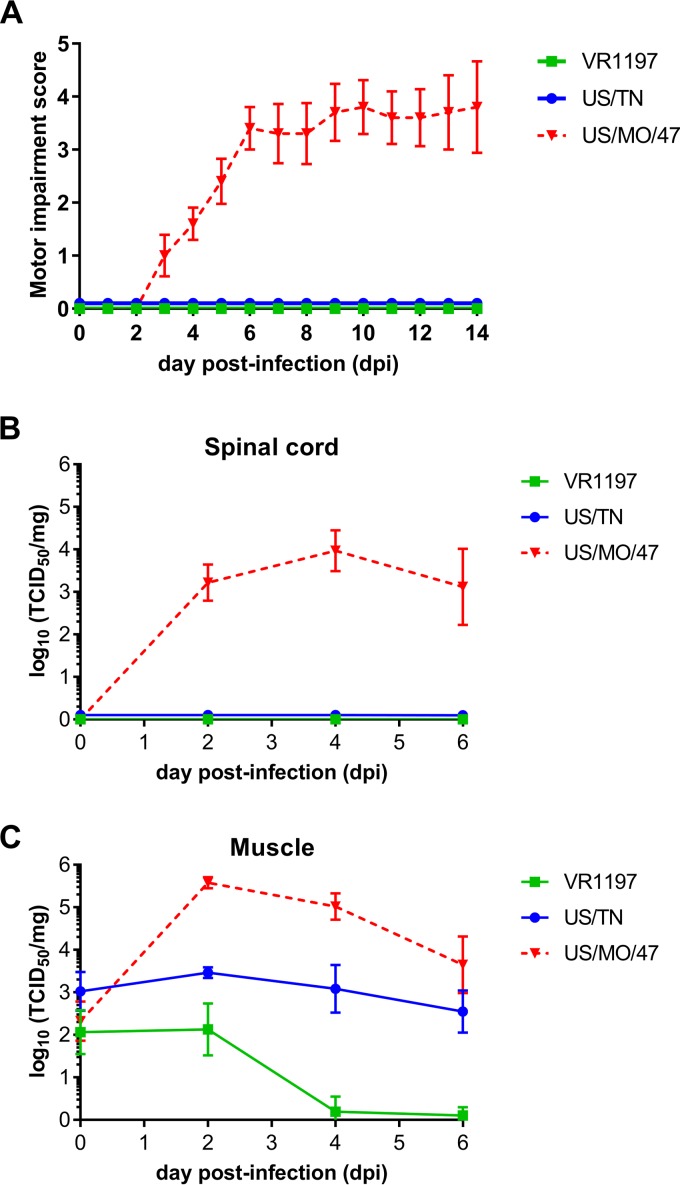
Differential motor impairment in mice following intramuscular injection. (A) Motor impairment was scored daily for 14 days post-intramuscular challenges with the indicated EV-D68 strains. None of the mice infected with US/TN or VR1197 developed signs of paralysis, whereas 100% of mice infected with US/MO/47 developed paralysis. Error bars represent standard error of the mean (SEM). (B and C) Viral titers from muscle and spinal cord titers were determined by TCID_50_ assay on samples taken at 0, 2, 4, and 6 days post-intramuscular infection. Error bars represent standard deviation (SD).

US/MO/47, US/TN, and VR1197 were assessed *in vivo* for their ability to cause paralysis and neuropathogenesis in 2-day-old outbred Swiss Webster mouse pups as previously described by Hixon et al. (21). Intramuscular injection of US/MO/47 resulted in limb paresis and paralysis in all mice injected (*n* = 10) as quantified by a motor impairment score (Fig. 2A). Most mice injected with US/MO/47 developed moderate to severe paralysis in both rear limbs. Paralysis always began in the injected hind limb and then spread to the contralateral hind limb in most animals, with rare spread of paralysis to the fore limbs. Quantification of the average motor impairment over time showed onset of weakness starting at approximately 4 days postinfection (dpi), with progressive worsening through 7 dpi, with the majority of mice continuing to have moderate to severe weakness in both hind limbs through the end of the observation period at 14 dpi. These data are consistent with previously published results on EV-D68-induced paralysis (21, 40). In particular, Hixon et al. (21) found that mice with paralysis by intramuscular injection of US/MO/47 analyzed histologically had corresponding cell death and viral antigen present in the anterior horns of the expected spinal cord regions. In contrast to US/MO/47, mice receiving intramuscular injection of US/TN (*n* = 11) or VR1197 (*n* = 10) failed to develop any signs of motor impairment during the 2-week observation period.

### Neurotropic EV-D68 can be detected in the spinal cords of paralyzed mice.

Intramuscular infection with US/MO/47 resulted in increased titers of infectious virus within mouse spinal cords paralleling the onset of motor impairment. Viral replication was first detected at 2 dpi in spinal cords (∼10^3^ TCID_50_/spinal cord), which corresponded with a rapid increase in viral titer within the muscle tissue (∼10^5^ TCID_50_/mg) of the injected limb (Fig. 2B). Viral titer remained detectable in both spinal cords (∼10^3^ TCID_50_/spinal cord) and muscle tissue (∼10^4^ TCID_50_/mg) at 6 dpi in US/MO/47-injected mice. In contrast, neither US/TN nor VR1197 produced detectable infection within mouse spinal cords. We observed sustained viral titers from US/TN within mouse muscle up to 6 dpi (∼10^3^ TCID_50_/mg), but no detectable spread of virus to spinal cord. VR1197 did not produce a sustained infection in mouse muscle, and the viral titer dropped to the limits of detection by 6 dpi.

### Replication kinetics of the recently circulating EV-D68 strain in the SH-SY5Y cell culture model.

**FIG 3:**
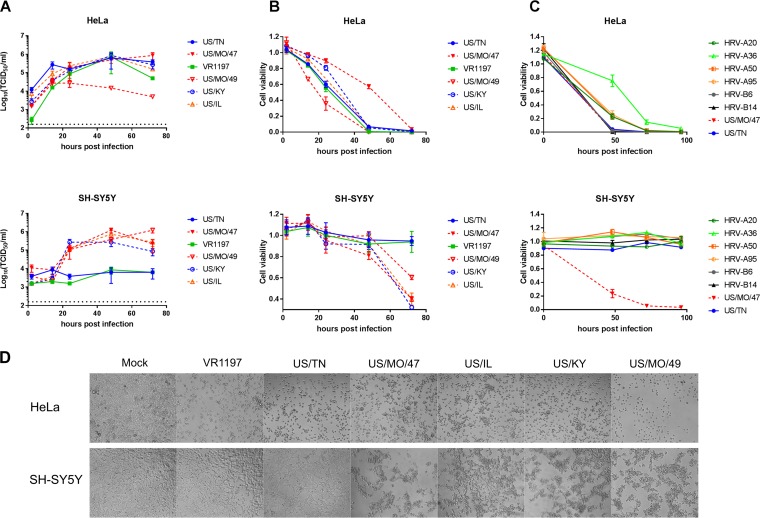
An expanded set of contemporary EV-D68 strains infect SH-SY5Y, but HRV strains do not. (A) HeLa and SH-SY5Y cells were infected with 6 different EV-D68 isolates at an MOI of 0.1. Cell culture lysates were collected at various time points, and the viral titer was determined by TCID_50_ in HeLa cells. Dotted black lines indicate the limit of detection. Error bars represent SEM from three biological replicates. (B) Similarly, cell viability was determined by quantifying the ATP content of the supernatant with CellTiter Glo (Promega) luminescence. Cell viability was calculated relative to mock-infected cultures. Error bars represent SEM from three replicates. (C) Six different human rhinovirus (HRV) strains and two EV-D68 strains were used to infect HeLa and SH-SY5Y cell cultures at an MOI of 0.1 and were visualized at 72 hpi. Cell viability was calculated as above. (D) Differential cytopathic effects of different EV-D68 isolates in HeLa and SH-SY5Y cells after infection with different EV-D68 isolates at an MOI of 0.1 before visualization at 72 hpi.

To further characterize the differential replication observed for diverse contemporary EV-D68 isolates and to test the SH-SY5Y cell infection mode, we obtained all additional commercially available strains of EV-D68. These included strains from the B1, B2, and D1 clades. Interestingly, all additional viral strains replicated in both HeLa and SH-SY5Y cells (Fig. 3A), including another strain from the B1 clade (US/MO/49), a strain from the newly defined D1 clade (US/KY), and a strain from the B2 clade (US/IL), replicating to a viral titer of ∼10^5^ TCID_50_/ml by 48 hpi. These three strains showed CPE after infection of SH-SY5Y cells (Fig. 3D**)**. All EV-D68 strains replicated at similar rates in HeLa cells.

To validate our qualitative CPE evaluation, we performed an independent assay of cell death using ATP content as determined by CellTiter Glo luminescence assay (Promega) as a surrogate for viable, intact cells. Using an MOI of 0.1, cell viability dropped after 12 hpi when HeLa cells were infected with every EV-D68 strain tested and continued to drop until the limit of detection was reached, between 48 and 72 hpi (Fig. 3B). In contrast, cell viability of infected SH-SY5Y cells, beginning at approximately 48 hpi, only dropped for strains where CPE was present. The results of the cell viability and CPE assays were reflective of the TCID_50_ data in HeLa and SH-SY5Y cells for the new panel. The cell viability assay was also performed at 37°C, which produced a similar replication pattern despite initial reports that EV-D68 grows poorly at 37°C (41). We observed similar rates of viral replication in HeLa and SH-SY5Y cells at both 33 and 37°C (see Fig. S3 in the supplemental material).

10.1128/mBio.01954-18.3 FIG S3 Cell viability in cells infected with EV-D68 at 37°C. Using replicate plates, cell viability was measured by quantifying ATP content as determined by CellTiter Glo (Promega) luminescence. Cell viability calculated relative to mock. Error bars represent SEM from four replicates. Download FIG S3, TIF file, 1.4 MB. Copyright © 2018 Brown et al. 2018 Brown et al. This content is distributed under the terms of the Creative Commons Attribution 4.0 International license.

### Human rhinovirus does not infect SH-SY5Y.

SH-SY5Y cells were tested for infectivity across a broad selection of HRV strains: two strains from the HRV-B lineage (HRV-B6 and HRV-B14) and four strains from the HRV-A lineage (HRV-A95, HRV-A50, HRV-A36, and HRV-A20). Using the cell viability assay with an MOI of 0.1, the number of viable cells dropped for all HRV strains in HeLa cells. However, in SH-SY5Y cells, no evidence of HRV infectivity was observed for any strain tested using either the cell viability assay (Fig. 3C) or visual inspection for CPE (see Fig. S4 in the supplemental material).

10.1128/mBio.01954-18.4 FIG S4 HRV does not infect SH-SY5Y. Six different HRV strains and two EV-D68 strains were used to infect HeLa and SH-SY5Y cell cultures grown in a 96-well plate at an MOI of 0.1 before visualization at 72 hpi. Download FIG S4, TIF file, 4.4 MB. Copyright © 2018 Brown et al. 2018 Brown et al. This content is distributed under the terms of the Creative Commons Attribution 4.0 International license.

### Differential infection by EV-D68 viral strains is the same in differentiated and undifferentiated SH-SY5Y cells and in primary human neurons.

To further characterize the differential replication capability of different EV-D68 strains, we differentiated SH-SY5Y using a well-established retinoic acid (RA) treatment protocol (42, 43), before virus infection, and confirmed differentiation by microscopic examination for morphological changes. We observed no difference in EV-D68 infectivity patterns between differentiated and undifferentiated SH-SY5Y cells. CPE observation (data not shown) and the viral replication rate (see Fig. S5 in the supplemental material) were similar compared to undifferentiated SH-SY5Y for all strains. All strains that could replicate in undifferentiated SH-SY5Y cells could also replicate in differentiated SH-SY5Y cells, and viral strains that could not replicate also did not replicate in differentiated SH-SY5Y cells. This demonstrates that EV-D68 strains are capable of infecting neuronal precursors and can also infect mature differentiated neuronal cells. Primary human fetal brain-derived neurons were cultured and infected with US/TN, VR1197, and US/MO/47 (see Fig. S6 in the supplemental material). Neurotropic and nonneurotropic EV-D68 strains showed the same infectivity pattern in SH-SY5Y. Using an MOI of 0.01, US/TN and VR1197 plated onto primary neuronal cells did not replicate and viral titers did not rise above the inoculation-level baseline. In contrast, an increase in viral titers was observed when the neurotropic strain US/MO/47-infected primary neuronal cells and reached peak titer ∼10^5^ TCID_50_/ml at 24 hpi.

10.1128/mBio.01954-18.5 FIG S5 EV-D68 virus titers in differentiated SH-SY5Y cells. Differentiated SH-SY5Y cells were infected with 6 different isolates of EV-D68 at an MOI of 0.1. Cell culture lysates/supernatants were collected at various time points. The viral titer was determined by TCID_50_ in HeLa cells. The dotted black line indicates the limit of detection. Error bars represent SEM from three biological replicates. Error bars represent SEM from three replicates. Download FIG S5, TIF file, 0.3 MB. Copyright © 2018 Brown et al. 2018 Brown et al. This content is distributed under the terms of the Creative Commons Attribution 4.0 International license.

10.1128/mBio.01954-18.6 FIG S6 EV-D68 strain growth in human postnatal cortical neurons. Human postnatal day 0 brain neurons were maintained to day 7 *in vitro* before infection with EV-D68 US/MO/47, US/TN, or VR1197 at an MOI of 0.01. Cell culture lysates/supernatants were collected at various times post-viral infection, and viral titers were measured using endpoint dilutions for growth in RD cells. The *x* axis indicates the limit of detection. Error bars represent standard deviation (SD) from three biological replicates. Download FIG S6, TIF file, 0.1 MB. Copyright © 2018 Brown et al. 2018 Brown et al. This content is distributed under the terms of the Creative Commons Attribution 4.0 International license.

### All EV-D68 strains generate virus when transfected into SH-SY5Y cells.

**FIG 4:**
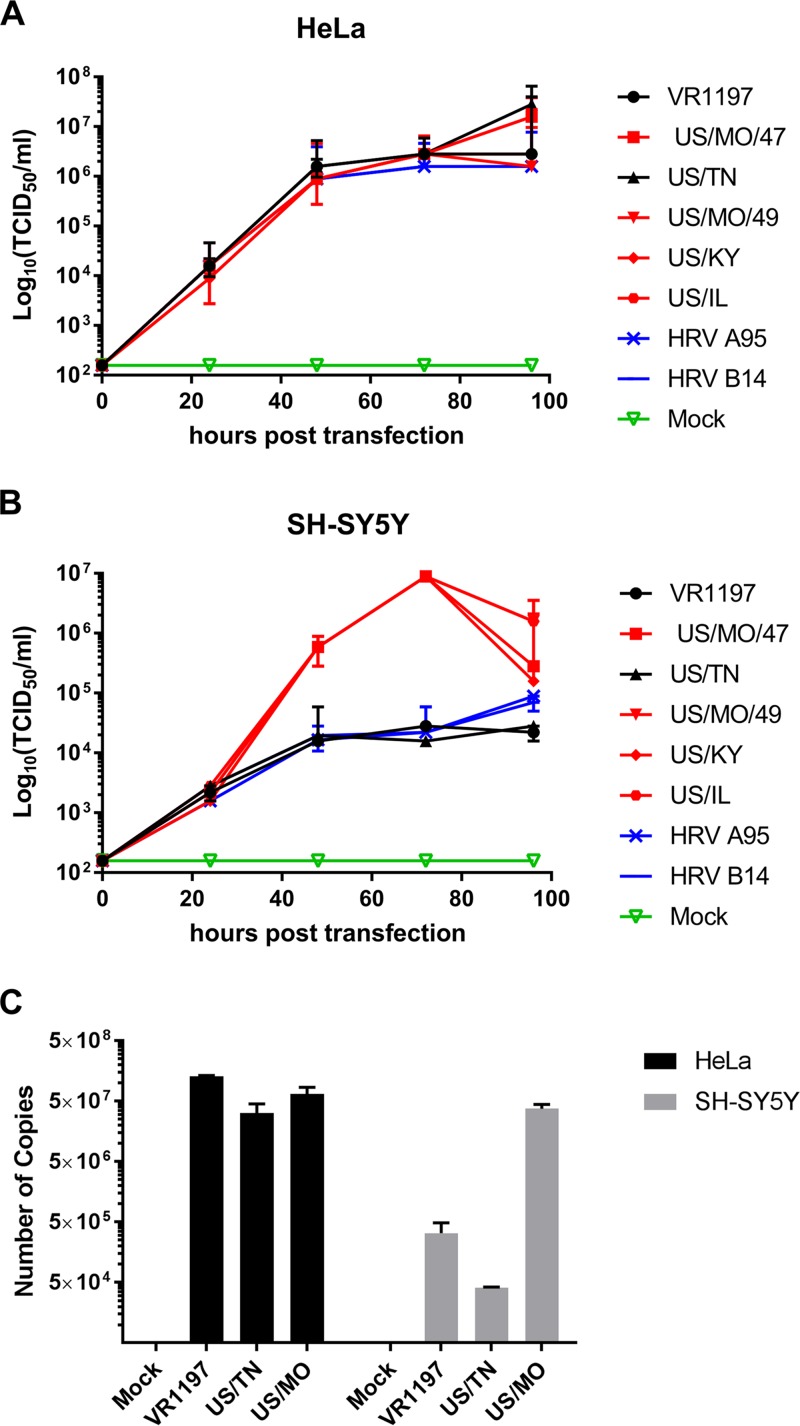
Cell binding and internalization is limiting for EV-D68 replication in neuronal cells. Replication of EV-D68 isolates following transfection of genomic RNA into (A) HeLa and (B) SH-SY5Y cells was evaluated. RNAs were purified from various HRV and EV-D68 virus stocks and used to transfect SH-SY5Y and HeLa cells. Cell culture lysates were collected at various time points and viral titer determined by TCID_50_ in HeLa cells. Error bars represent SEM from three biological replicates. (C) EV-D68 US/MO/47, US/TN, or VR1197 virus preparations were incubated with HeLa or SH-SY5Y cells for 2 h at an MOI of 1, and virus attachment was subsequently measured by RT-qPCR. Copy number was calculated relative to a cDNA standard curve. Error bars represent SEM from three biological replicates.

To determine if virus cell entry may be responsible for restricting virus replication in SH-SY5Y for some isolates, full-length genomic RNA from each of the EV-D68 isolates was transfected into the cytoplasm of SH-SY5Y cells and virus production was measured. RNA transfection into HeLa cells resulted in a viral infection and replication pattern similar to intact virus infections at an MOI of 0.1, with viral titers peaking at ∼10^7^ TCID_50_/ml for all EV-D68 strains tested. In contrast to what we observed using standard infection assays, all tested D68 strains generated virus following RNA transfection into SH-SY5Y cells. However, the viral titer peaked at 10^4^ TCID_50_/ml 48 h after transfection of SH-SY5Y for viral strains that could not infect SH-SY5Y cells using intact virions (Fig. 4A and B), whereas the viral titer continued to increase until saturation at approximately 10^7^ TCID_50_/ml following transfection of SH-SY5Y with strains that could infect SH-SY5Y cells.

### EV-D68 binding efficiency to SH-SY5Y is significantly increased for neurotropic strains.

To determine if viral attachment is the bottleneck to viral entry for the nonneurotropic phenotype observed, we performed a virus-cell binding assay (44). EV-D68 strains at an MOI of 1 were incubated with either HeLa cells or SH-SY5Y cells at 4°C for 2 h. Upon washing cells to remove any unbound virus, cell-associated viral copy numbers were determined by real-time quantitative PCR (RT-qPCR). Threshold cycle (*C_T_*) values were obtained, and viral copy numbers were calculated based on a standard curve **(**Fig. 4C**)**. HeLa cells incubated with EV-D68 strains VR1197, US/TN, and US/MO/47 showed substantial virus retention and did not show a significant difference in binding activity. In contrast, >100-fold more copies of US/MO/47 were bound to SH-SY5Y compared to US/TN and VR1197.

## DISCUSSION

**FIG 5:**
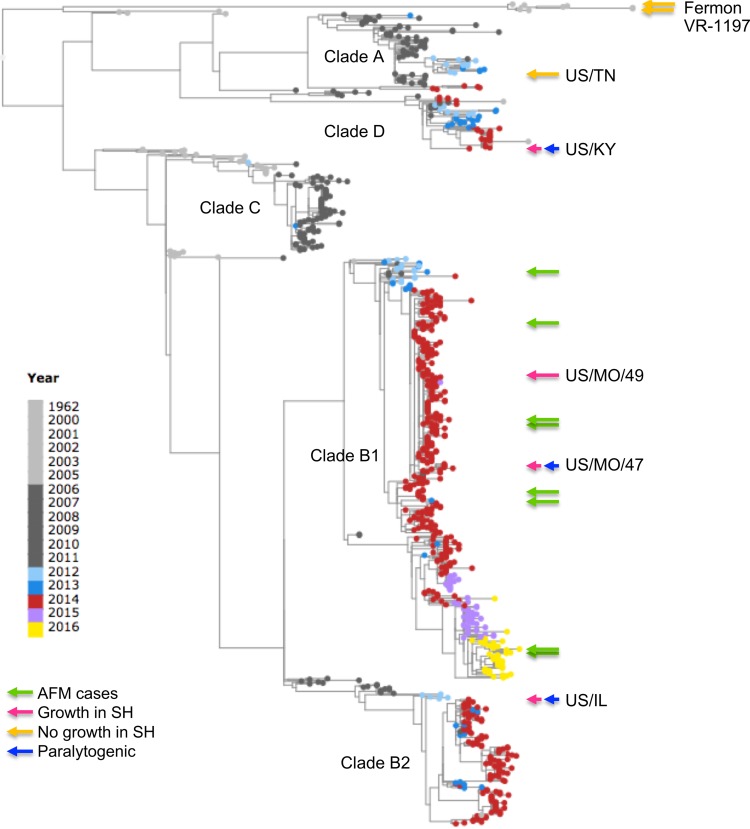
Phylogenetic tree of EV-D68 isolates based on VP1 sequences. VP1 nucleotide sequences of >900 nt were retrieved from the ViPR site (https://www.viprbrc.org/brc/home.spg?decorator=picorna_entero) on 24 July 2017. Sequences were aligned using the MUSCLE algorithm and sequences showing poor alignments were removed. A phylogenetic tree was computed using RaXML (bootstrap replicates of 100) and then visualized using Archaeopteryx.js via the ViPR site. Clade classifications are based on bootstrap values of 100%. AFM-associated isolates are marked with a green arrow. EV-D68 isolates used in this study are labeled with either a pink (growth in SH-SY5Y cells) or orange (no growth in SH-SY5Y cells) arrow. Isolates that are paralytogenic in mice (56) are labeled with a blue arrow.

Here we report on the differential infectivity between various contemporary and historical EV-D68 strains in SH-SY5Y as measured by viral replication, cell viability, CPE, and immunofluorescence. The clade specificity of neuropathogenesis previously reported (10, 22, 26–28, 33, 34) between contemporary and historical strains is observed in the neurotropism in SH-SY5Y cells (Fig. 5). Among the EV-D68 strains used in this study, those from clades B1, B2, and D1 were able to infect SH-SY5Y cells, whereas those from clade A and other historical strains could not (45). We also showed that this differential growth is at least partly due to differential viral entry, as all strains can replicate and produce viral progeny after transfection.

To determine the most appropriate tissue culture model system, we explored the gene expression by RNA sequencing and showed that transcripts from SH-SY5Y cells are enriched for neuronal-specific genes relative to HeLa and HTB10 cells. This agrees with several other groups which have shown the expression of specific neurological marker genes in SH-SY5Y cells (42, 46, 47). Of note for this study, SH-SY5Y also showed relatively high expression of the ISL1 gene, which is expressed in all postmitotic motor neurons and is required for various aspects of motor neuron development (48). This neuronal cell line has successfully been used as a model to study the *in vitro* neuropathogenic effects of different viruses, notably, as a model for paralytic enteroviral isolates, including EV-A71 and poliovirus (36, 49–55). Numerous other viruses associated with neurological symptoms can infect SH-SY5Y, including: Japanese encephalitis virus (56), human immunodeficiency virus (57), human cytomegalovirus (58), varicella-zoster virus (59), chikungunya virus (60), mumps virus (61), dengue virus (62), Zika virus (63, 64), and rabies virus (65), suggesting that SH-SY5Y is an excellent model system for neurotropic viruses.

We found support of the biological relevance of SH-SY5Y cells as a cell culture model by observing the same infectivity pattern *in vitro* with other neuronal cell cultures. SH-SY5Y cells are capable of being differentiated using retinoic acid, which leads to more neuron-specific morphology and gene expression. These differentiated cells are characterized by the formation of extensive neurites, as well as the induction of neuron-specific enzymes, receptors, and neurotransmitters (42). Differentiated SH-SY5Y cells have been used as models for neuron-virus interactions (66) including EV-A71 (54, 55), chikungunya virus (60), and varicella-zoster virus (59). Our results show that both differentiated and undifferentiated SH-SY5Y can be infected with neurotropic strains of EV-D68, suggesting neurotropic strains of EV-D68 can invade both immature and mature neuronal cells. Primary human fetal brain-derived neurons have also been used to study infectivity patterns of neurotropic viruses as well as the central nervous system (67, 68). We also observed a similar EV-D68 replication pattern in human primary postnatal neurons. Interestingly, despite initially reported as growing poorly at 37°C (41), we observed similar rates of EV-D68 replication in SH-SY5Y cells at both 33 and 37°C, which would likely be a more biologically relevant temperature for paralytic infections at the core body temperature rather than the lower temperature of the upper respiratory system that supports more routine respiratory infection.

EV-D68 is closely related to human rhinovirus (HRV) within the Picornaviridae family (69). Frequent coinfection in patients and cross-reactivity of quantitative PCR-based screening had led to misdiagnosis of EV-D68 infection as HRV infection prior to the 2014 outbreak (70, 71). Despite the similarity in respiratory symptoms, HRV lacks any association with the neurological symptoms of other enteroviruses, such as EV-D68 and EV-A71 (69). Indeed, despite their similarity to EV-D68, none of the 6 HRV strains could infect SH-SY5Y cells.

We hypothesized that the reason for differential replication in SH-SY5Y cells of EV-D68 strain relates to different viral entry capabilities. We used RNA transfection to deliver infectious RNA to the cytoplasm, bypassing natural viral entry mechanisms during an infection. All EV-D68 strains generated virus following RNA transfection, but in some cases, viral titers plateaued at a relatively lower level, suggesting that only a single round of replication had occurred. HRV RNA transfected into SH-SY5Y cells produced a similar result. Our interpretation is that differences in the sequence and structure of viral capsid proteins are responsible for the differential infectivity in SH-SY5Y cells by EV-D68 strains and that viral entry is what prevents HRV, US/TN, and the historical strains from infecting SH-SY5Y cells. It has recently been reported that a chimeric swap mutant exchanging the viral capsid from EV-D68 VR1197 and a neurotropic EV-D94 strain capable of replication in SH-SY5Y cells results in a loss of infectivity in SH-SY5Y cells (45). This result further supports our conclusion that viral entry mediated by the capsid is the cause of the observed differential neurotropism. We performed a virus-cell binding assay (44, 72) that further supports this conclusion. Approximately 100 times more copies of viral RNA were detected, indicating more virus particles bound to cells when US/MO/47 was incubated with SH-SY5Y cells compared to US/TN and VR1197. This indicates that the binding affinity of US/MO/47 to SH-SY5Y neuronal cells is greater than that of nonneurotropic strains, suggesting that virus binding could be limiting for D68 replication in neuronal cells. However, viral entry was not blocked and therefore may be measured in addition to viral binding. Specific genetic residues may be the cause of this differential neurotropism. A comparative analysis using infectious clones bearing specific polymorphisms will likely be needed to establish the determinants of neurotropism in SH-SY5Y cells. In particular, the 2014 outbreak B1 substitutions in VP1/98A, VP1/148V, VP1/280K, VP1/290S, VP1/308N, and VP2/222T are all located on the virion surface and could be directly involved in virus-host cell attachment and would be good candidates to evaluate.

Our results closely correlate with the differential paralytic myelitis caused by EV-D68 in mice suggesting that infectivity in SH-SY5Y cells may be an effective proxy for neuropathogenesis (Fig. 5). Multiple contemporary EV-D68 strains that have been shown to cause paralytic myelitis in mice were also neurotropic in SH-SY5Y cells (21). However, not all of the contemporary strains that exhibit *in vitro* neurotropism (e.g., US/CA/14-4231) have been shown to cause myelitis in mice, suggesting there could be further host- or species-specific factors that also contribute to the development of myelitis when infected with a neurotropic EV-D68 strain (21). The historical, nonneurotropic strains that are nonparalytic in mice did not grow in SH-SY5Y cells. In particular, the contemporary EV-D68 strain, US/TN, which was nonneurotropic in SH-SY5Y cells and not previously reported by Hixon et al. (21), failed to produce paralysis in mice and could not be found in mouse spinal cord tissue.

US/TN appeared to replicate at a low level within mouse muscle tissue, indicating lack of paralysis was not due to an inability to infect the mice. Interestingly, one alternative hypothesis for how EV-D68 might cause paralysis is through myositis, not myelitis (73). This is based on the observation that an intranasal-adapted EV-D68 stain used to intranasally inoculate immunodeficient mice (AG129) causes paralysis with the absence of myelitis. Our observed increased replication in muscle by US/MO/47 relative to US/TN and VR1197 (Fig. 2) suggests that may be possible. However, the lack of myelitis with the intranasal-adapted EV-D68 strain may be due to different genetic residues, arising from the 30 passages of US/MO/49 used to generate the intranasal-adapted EV-D68 strain, potentially disabling viral entry to neuronal cells. Of note, Swiss Webster mice challenged intranasally with EV-D68 US/MO/47 appear to develop myelitis as detected by staining for viral antigen and RT-PCR for virus in spinal cord tissue (21). Testing the neurotropism of the intranasal-adapted EV-D68 stain (73) via SH-SY5Y cells would help elucidate if there are potentially multiple mechanisms by which EV-D68 causes paralysis.

The infectivity pattern in SH-SY5Y cells, along with the agreement in primary postnatal neurons, supports the mouse model reported by Hixon et al. (21) and validates the results in the SH-SY5Y human cell line. This is significant because, while useful, mouse models are costly and introduce potential caveats, such as transcriptional factor differences in mice versus humans (74–76), and there is debate whether the mouse model recapitulates human conditions (77, 78). Evidence presented here supports the validity of the mouse model.

In conclusion, we present a differential neuronal infectivity phenotype between contemporary and historical EV-D68 strains. Permissible infection of SH-SY5Y cells mimics the paralysis pattern reported in animal models. The high-throughput nature of tissue culture models will allow for rapid screening of novel viral strains and recombinant viruses to elucidate the genetic determinants of neurotropism and potential antiviral therapies. This can enable identification of EV-D68 signatures of virulence responsible for neural infection and potentially neurological disease and avoids the cost associated with large-scale screening using animal models. Further, this supports the theory of a causal link between AFM in humans and EV-D68.

## MATERIALS AND METHODS

### Cell culture.

HeLa cells (ATCC) were maintained in Dulbecco’s modified Eagle’s medium (DMEM; Gibco) supplemented with 10% fetal bovine serum (FBS; HyClone). HTB10 (ATCC) cells were maintained in DMEM supplemented with 10% FBS and nonessential amino acids (Gibco). SH-SY5Y (CLR-2266; ATCC) cells were maintained in a 1:1 mixture of DMEM and F-12 (Gibco) medium supplemented with 10% FBS. To differentiate SH-SY5Y cells, an ∼50% confluent flask of SH-SY5Y cells had medium replaced with 1:1 mixture of DMEM and F-12 (Gibco) medium supplemented with 3% FBS and 10 µM retinoic acid (RA [Sigma]) (53). After 3 days of exposure to RA, the morphology of cells was evaluated, and cells were passaged for further use. Morphology and cytopathic effect were evaluated using an inverted microscope. Human postnatal day 0 (P0) brain neurons were purchased from ScienCell (catalog no. 1520) and plated at a density of 40,000 neurons/well on poly-d-lysine-coated 96-well plates (68). The neurons were maintained in ScienCell neuronal growth medium with penicillin-streptomycin (catalog no. 1520) at 37°C in 5% CO_2_ until day *in vitro* (DIV) 7, by which time the neurons had well-established neurites.

### Virus stock preparation.

EV-D68 stocks were prepared by infecting HeLa or rhabdomyosarcoma (RD) cells (ATCC) at 33°C in 5% CO_2_ until CPE was observed. Cell debris was removed by centrifugation, and titers were determined in a standard 50% tissue culture infective dose (TCID_50_) assay and calculated by the Spearman-Kärber method. The source of each strain is detailed in Table S1 in the supplemental material.

10.1128/mBio.01954-18.7 TABLE S1 List of strains used in this study. Download Table S1, DOCX file, 0.1 MB. Copyright © 2018 Brown et al. 2018 Brown et al. This content is distributed under the terms of the Creative Commons Attribution 4.0 International license.

### Replication kinetics of EV-D68.

The replication kinetics for HeLa, A549, HTB10, SH-SY5Y, and differentiated SH-SY5Y cells were evaluated in a high-throughput manner. Viral replication kinetics were measured from sets of flat-bottom 96-well plates. Sets of plates corresponding to the number of desired time points in an experiment were infected at the same initial time, using distinct 96-well plates for each time point. Infected plates were incubated at 33 to 34°C with 5% CO_2_ until the designated time point, when each corresponding plate was placed in a −80°C freezer until the entire time course was completed. Mock-infected wells adjacent to each condition demonstrated that no contamination occurred across wells. After 2 h, high-MOI plates (0.1 and 1) were washed three times with phosphate-buffered saline (PBS), and the 2-h time point plate was frozen to determine the background levels of virus present, since 2 h is long enough for EV-D68 entry but not long enough for replication. After three freeze-thaw cycles, the viral titers from 10-fold serial dilutions of each sample were evaluated using a 50% TCID_50_ assay on HeLa cells. Plates were scored after adding 100 µl of crystal violet fixative per well followed by 1 h of incubation at room temperature and washing to remove unbound dye. Crystal violet fixative was prepared by adding 5 g crystal violet (Sigma) and 8.5 g sodium chloride (Sigma) to 50 ml formaldehyde, 260 ml ethanol, and 690 ml deionized water.

For replication kinetics in human postnatal neurons, day *in vitro* (DIV) 7 neurons were infected with EV-D68 US/MO/47, US/TN, or VR1197 at an MOI of 0.01. Infection medium was left on the cells for the duration of the experiment to minimize loss of cells from multiple rinses due to low cell adhesion. Cell culture supernatant and lysate were collected at 0, 6, 12, 24, 48, and 72 h, with three biological replicates collected per time point for each viral strain. Lysate was serially diluted 10-fold from 1 (raw lysate) to 10^−6^ RD cells at 33°C and evaluated using a 50% TCID_50_ assay.

### Immunostaining.

HeLa and SH-SY5Y cells were grown to 50% to 70% confluence on coverslips in a 24-well plate and infected with EV-D68 strains at an MOI of 1.0. Mock-infected cells serve as a negative control. Coverslips were incubated at 34°C in 5% CO_2_ for 18 h and then fixed with 4% paraformaldehyde (PFA) and stored at 4°C. The coverslips were washed with PBS, and cells were permeabilized with 0.1% Triton-X for 10 min. The coverslips were blocked with 2% bovine serum albumin in PBS for 1 h. The cells were incubated with rabbit polyclonal anti-VP1 of EV-D68 (GeneTex) at a final concentration of 4 µg/ml overnight at 4°C, washed 3 times, and then incubated with a secondary goat anti-rabbit rhodamine red-X (Thermo Fisher) at a final concentration of 1 µg/ml for 30 min. To visualize nuclei, DAPI (4′,6-diamidino-2-phenylindole) stain was added to the second of three washes. The wells were visualized on an Axioskop 2 Plus (Zeiss) fluorescence microscope using DAPI and rhodamine filters. Images were taken with an AxioCam MRc5 (Zeiss) camera using AxioVision software. All images for a particular filter were taken under identical exposure conditions.

### Mouse infections with EV-D68.

Animal experiments were performed in an AAALAC-accredited animal facility under IACUC protocol B-34716(03)1E at the University of Colorado. Pregnant female Swiss Webster mice were ordered from Envigo and kept in standard housing until the pups were born. At postnatal day 2, the dam and pups were transferred the biosafety level 2 (BSL2) region of the animal facility. P2 Swiss Webster mouse pups were then inoculated with 10^6.8^ TCID_50_/ml virus in 10 μg by intramuscular injection into the left medial hind limb (21). Mouse pups of both sexes were randomized to treatment conditions before virus inoculation.

### Motor impairment scoring.

Mice were monitored daily for 14 days. To assess paralysis, mice were removed from the cage and observed moving on a flat surface for several minutes, during which each limb was given a motor impairment score: 0, no motor impairment; 1, mild motor impairment, ataxia, or decreased movement present and toe/knuckle walking; 2, moderate impairment, profound ataxia, and limited movement of limb; and 3, severe impairment, no movement in limb, and limb is non-weight bearing. The final motor impairment score for each day was achieved by summing the score for each limb.

### Mouse tissue collection.

Mouse pups were sacrificed by decapitation for collection of muscle and spinal cord tissue. Spinal cords were removed as previously described (21, 40). Muscle tissue was collected from the inoculated limb (with the goal of obtaining as much muscle tissue possible from the anterior and posterior thigh and gastrocnemius). Both tissues were collected into BeadBug tubes containing inert ceramic bead and 0.3 ml of ice-cold, sterile PBS. Tissues were lysed mechanically on a BeadBug tissue homogenizer for 45 s at 2,800 rpm and stored at −80°C. After thawing, tissue samples were spun at 2,700 *× g* for 1 min to remove tissue chunks from the lysate. Lysate was serially diluted 10-fold from 1 (raw lysate) to 10^−6^ and plated in a standard TCID_50_ assay to determine the final viral titer. To get the final titer per whole spinal cord, the TCID_50_/ml was multiplied by 0.3 ml. To get the final muscle titer per milligram of tissue, the TCID_50_/ml was multiplied by 0.3 ml and divided by the weight of tissue collected. Samples that were below the limit of detection were graphed at zero.

### Cell ATP/viability assay.

Cells were cultured and evaluated in the same manner as in the viral replication kinetics assays. ATP levels were measured using the CellTiter-Glo luminescent cell viability assay kit (catalog no. G7570; Promega), and cell viability was calculated relative to that of the mock control. To preserve the ATP levels so that each time point could be evaluated concurrently, cell supernatant was removed and cells were frozen at −80°C. Once the time series was completed, all plates were removed and room temperature medium was added to each plate. Upon stabilization at room temperature for 20 min, the manufacturer’s protocol was followed. We validated this deviation from the manufacturer’s protocol by confirming the linearity of the assay across the active range of the study.

### RNA sequencing.

In order to explore the use of SH-SY5Y as an appropriate neuronal cell model, we used RNA sequencing to obtain a comprehensive view of the genes expressed in the SH-SY5Y, HeLa, and HTB10 cell lines. To prepare cells for RNA sequencing, 10^4^ cells were grown in a 96-well plate for 24 h in quadruplicate before being washed and resuspended in 10 µl of cell lysis buffer (0.2% Triton X-100, 2 U/µl RNase inhibitor, 1:2,000,000 dilution of ERCC spike-in RNAs [Life Technologies]) per well. Full-length cDNA was amplified using the SmartSeq2 protocol optimized in our laboratory (79, 80) before Nextera XT library preparation and sequencing on a NextSeq500 with 2 × 150 paired-end reads. After adapter/primer trimming using the Trimmomatic tool (http://www.usadellab.org/cms/?page=trimmomatic), trimmed sequencing reads were mapped to transcripts derived from the human reference genome (GRCh37), and gene expression levels (transcripts per million reads [TPM]) were estimated using the RSEM package (81).

### RNA purification and transfection.

RNA used for transfections was purified from viral stocks grown in HeLa cells. Purification was performed using QIAamp MinElute Virus Spin kit (Qiagen) according to the manufacturer’s instructions; final RNA concentrations were approximately 100 ng/µl. In 12-well plates, selected cell cultures were seeded and grown. According to the manufacturer’s instructions, 200 ng of RNA was used with 2 µl of each reagent in the *Trans*IT-mRNA transfection kit (Mirus) to perform a transfection.

### Virus-cell binding assay and RT-qPCR.

In a 12-well plate, approximately 3 × 10^5^ HeLa cells or SH-SY5Y cells were incubated with EV-D68 virus preparations for 2 h at 4°C using an inoculum MOI of 1.0 in a volume of 1 ml. After incubation, plates, were washed 3 times with 1 ml of PBS (Gibco), based on a protocol described previously (44). RNA was extracted using QIAamp MinElute Virus Spin kit (Qiagen) according to the manufacturer's instructions. The primers used to amplify EV-D68 were 5′ -TAACCCGTGTGTAGCTTGG-3′ and 5′-ATTAGCCGCATTCAGGGGC-3′, which are specific to the 5′ untranscribed region (UTR) of EV-D68 and conserved among the strains used. One-step RT-qPCR was performed using LightCycler EvoScript RNA SYBR green I Master kit (Roche) according to the manufacturer’s instructions and quantified using a LightCycler 480 Instrument II. RNA copies were calculated based on a cDNA standard curve.
