# Supplementary figures and images for: Contemporary Circulating Enterovirus D68 Strains Have Acquired the Capacity for Viral Entry and Replication in Human Neuronal Cells
Source: mBio. 2018 Oct 16;9(5):e01954-18. doi: 10.1128/mBio.01954-18 (PMC6191546; doi:10.1128/mBio.01954-18)

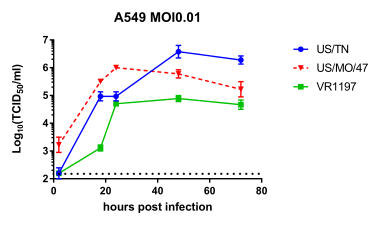

Supplement: FIG S1 [file mbo005184112sf1.tif]

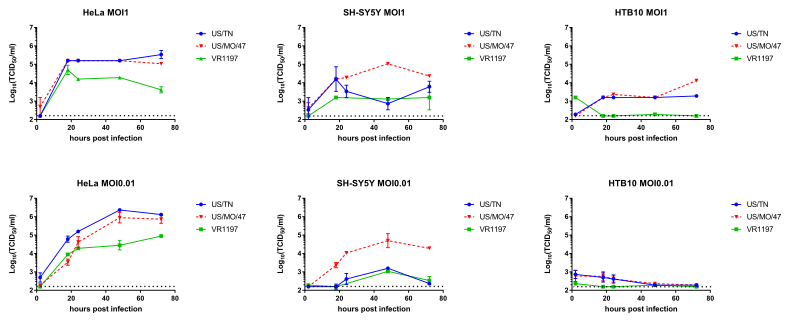

Supplement: FIG S2 [file mbo005184112sf2.tif]

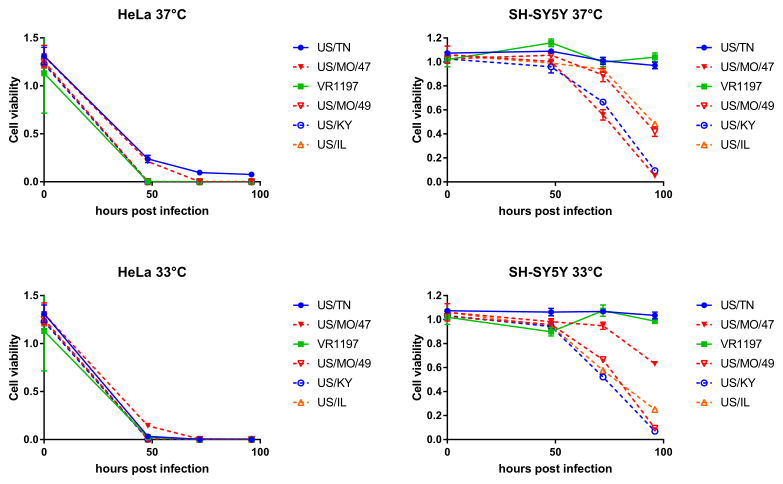

Supplement: FIG S3 [file mbo005184112sf3.tif]

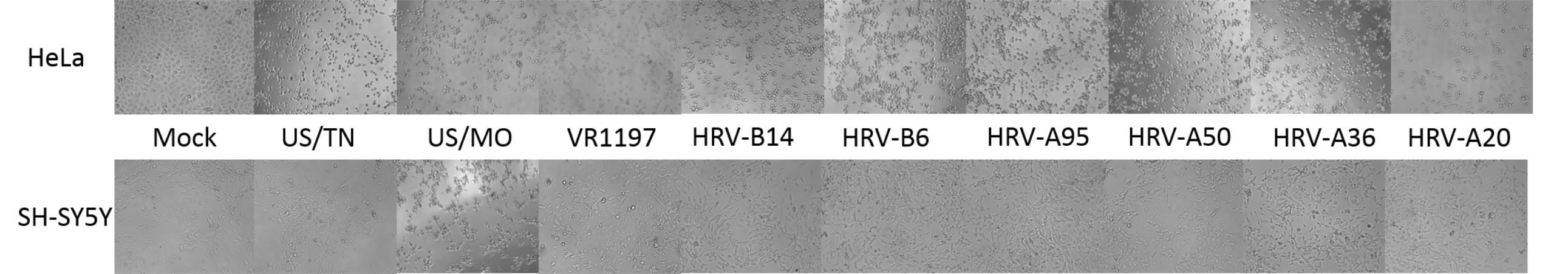

Supplement: FIG S4 [file mbo005184112sf4.tif]

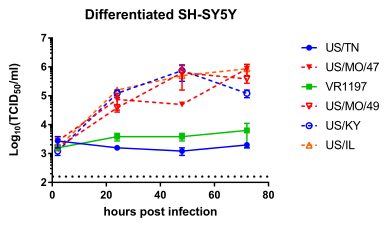

Supplement: FIG S5 [file mbo005184112sf5.tif]

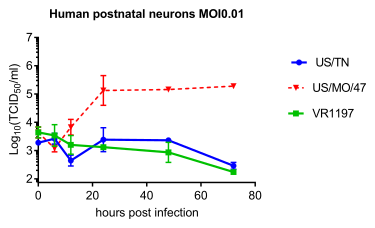

Supplement: FIG S6 [file mbo005184112sf6.tif]
