# Supplementary material for: Contemporary Circulating Enterovirus D68 Strains Have Acquired the Capacity for Viral Entry and Replication in Human Neuronal Cells
Source: mBio. 2018 Oct 16;9(5):e01954-18. doi: 10.1128/mBio.01954-18 (PMC6191546; doi:10.1128/mBio.01954-18)
Supplement: TABLE S1 [file mbo005184112st1.docx]

**S1 Table: List of strains used in this study.**

| Species | Strain name | Abbreviation | D68 clade | Accession # | Source |
| --- | --- | --- | --- | --- | --- |
| Enterovirus D68 | USA/N0051U5/2012 | US/TN | A | KT347280 | Dr. Tina V. Hartert |
| Enterovirus D68 | US/MO/14-18947 | US/MO/47 | B1 | KM851225 | ATCC VR-1823 |
| Enterovirus D68 | ATCC VR-1197 | VR-1197 | Fermon | KT725431 | ATCC VR-1197 |
| Enterovirus D68 | US/IL/14-18952 | US/IL | B2 | KM851230 | ATCC VR-1824 |
| Enterovirus D68 | US/KY/14-18953 | US/KY | D1 | KM851231 | ATCC VR-1825 |
| Enterovirus D68 | US/MO/14-18949 | US/MO/49 | B1 | KM851227 | BEI NR-49130 |
| Human Rhinovirus A | 15-CV19 | HRV-A20 | N/A | JN614993 | ATCC VR-495 |
| Human Rhinovirus A | SF-998 | HRV-A95 | N/A | FJ445170 | ATCC VR-1301 |
| Human Rhinovirus A | A2#58 | HRV-A50 | N/A | FJ445135 | ATCC VR-517 |
| Human Rhinovirus A | 342 H [V-171-001-021] | HRV-A36 | N/A | JF781497 | ATCC VR-1146 |
| Human Rhinovirus B | 1059 | HRV-B14 | N/A | NC_001490 | ATCC VR-284 |
| Human Rhinovirus B | Thompson | HRV-B6 | N/A | JN614996 | ATCC VR-486 |
